# Cisplatin radiosensitizes radioresistant human mesenchymal stem cells

**DOI:** 10.18632/oncotarget.21214

**Published:** 2017-09-23

**Authors:** Alexander Rühle, Ramon Lopez Perez, Christin Glowa, Klaus-Josef Weber, Anthony D. Ho, Jürgen Debus, Rainer Saffrich, Peter E. Huber, Nils H. Nicolay

**Affiliations:** ^1^ Department of Molecular and Radiation Oncology, German Cancer Research Center (DKFZ), 69120 Heidelberg, Germany; ^2^ Heidelberg Institute for Radiation Oncology (HIRO), National Center for Radiation Research in Oncology, 69120 Heidelberg, Germany; ^3^ Department of Radiation Oncology, Heidelberg University Hospital, 69120 Heidelberg, Germany; ^4^ Department of Hematology and Oncology, Heidelberg University Hospital, 69120 Heidelberg, Germany

**Keywords:** mesenchymal stem cells, cisplatin, radiotherapy, DNA double-strand breaks, radiosensitization

## Abstract

Cisplatin-based chemo-radiotherapy is widely used to treat cancers with often severe therapy-associated late toxicities. While mesenchymal stem cells (MSCs) were shown to aid regeneration of cisplatin- or radiation-induced tissue lesions, the effect of the combined treatment on the stem cells remains unknown. Here we demonstrate that cisplatin treatment radiosensitized human bone marrow-derived MSCs in a dose-dependent manner and increased levels of radiation-induced apoptosis. However, the defining stem cell properties of MSCs remained largely intact after cisplatin-based chemo-radiation, and stem cell motility, adhesion, surface marker expression and the characteristic differentiation potential were not significantly influenced. The increased cisplatin-mediated radiosensitivity was associated with a cell cycle shift of MSCs towards the radiosensitive G2/M phase and increased residual DNA double-strand breaks. These data demonstrate for the first time a dose-dependent radiosensitization effect of MSCs by cisplatin. Clinically, the observed increase in radiation sensitivity and subsequent loss of regenerative MSCs may contribute to the often severe late toxicities observed after cisplatin-based chemo-radiotherapy in cancer patients.

## INTRODUCTION

Ionizing radiation (IR) damages cells primarily by inducing DNA lesions, resulting in apoptosis, cell cycle arrest or mutagenesis. Cellular radiation sensitivities vary considerably and may be influenced by the cells’ DNA repair capacity or the presence of modifying agents including oxygen or cytotoxic drugs [1–4].

The anti-cancer agent cisplatin is widely used together with IR, and several large trials have demonstrated the safety and efficacy of cisplatin-based chemo-radiation regimens for various cancers [5–8]. Cisplatin binds to DNA strands and creates intra-strand and inter-strand crosslinks, thereby inhibiting DNA replication and transcription [9]. Cisplatin treatment can cause severe adverse effects in many organs, including bone marrow, nervous system, inner ear and kidneys, and cisplatin-induced renal damage commonly constitutes the dose-limiting toxicity [10, 11]. Cisplatin has been shown to influence radiation sensitivities of various cell types *in vitro*, and several potential mechanistic explanations have been provided, concerning radiation-induced increases in cisplatin uptake, efficient blockade of DNA repair or prolonged cell cycle arrest [12]. However, the exact mechanism underlying the radiosensitization of cisplatin remains incompletely understood.

Mesenchymal stem cells (MSCs) constitute a heterogeneous population of multipotent stromal cells that are found in various tissues like bone marrow, adipose and vascular tissues, placenta or umbilical cord [13–15]. MSCs can be identified by a combination of functional and molecular characteristics, including their ability to adhere to plastic surfaces, their differentiation potential and a characteristic surface marker expression [16, 17]. Due to their immunomodulatory and differentiation capabilities, MSCs have been investigated as a potential means of repairing tissue damage, and preclinical data suggest beneficial effects of MSC-based treatments for the therapy of radiation- or cisplatin-induced tissue injuries [18]. However, the effects of clinically relevant combination treatments on MSCs remain completely unknown.

## RESULTS

### Cisplatin radiosensitizes MSCs in a dose-dependent manner

**Figure 1:**
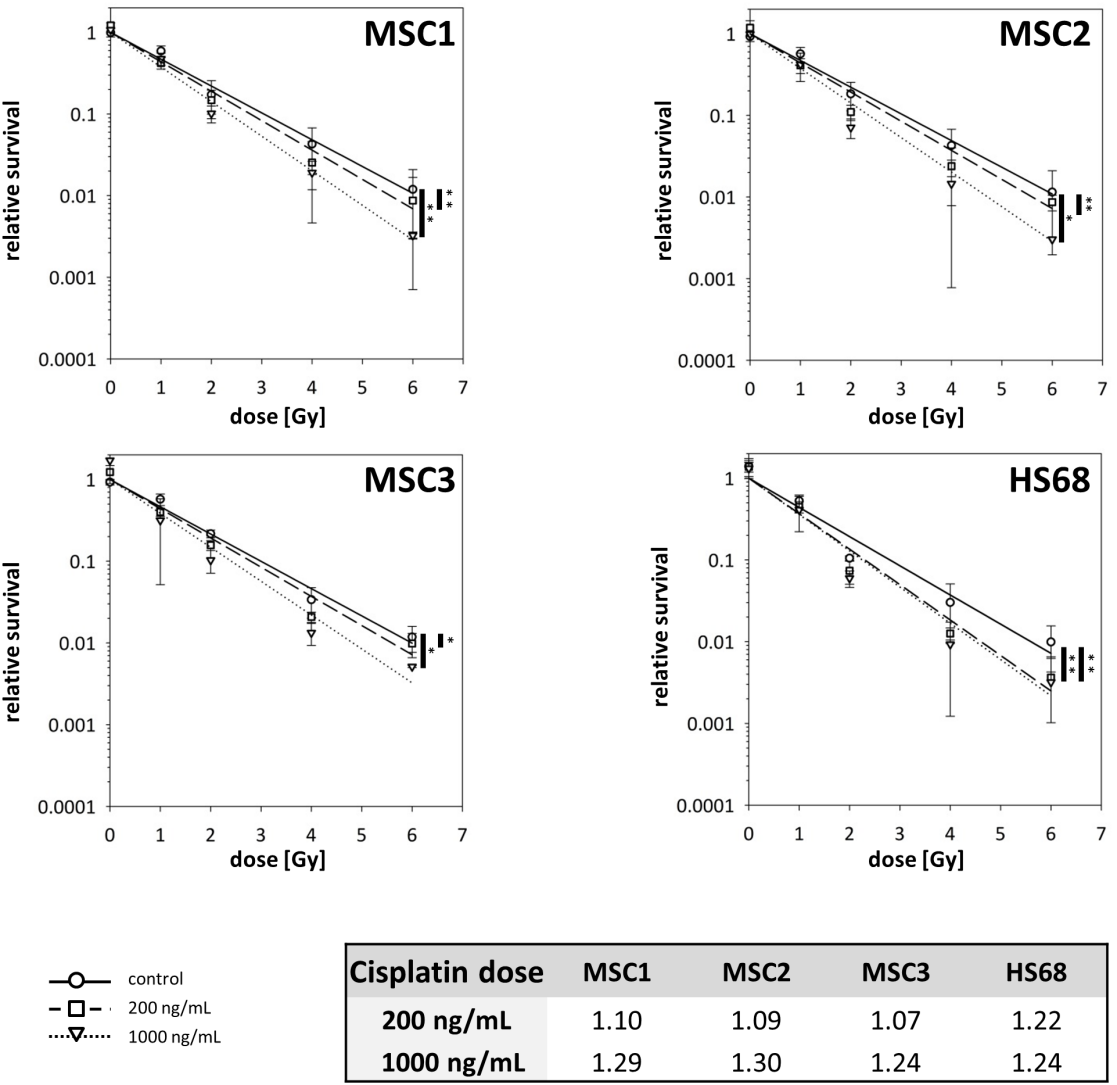
Cisplatin pre-treatment radiosensitizes MSCs Clonogenic survival assays for MSCs and HS68 fibroblasts pre-treated with 200ng/mL or 1000ng/mL cisplatin 48 prior to irradiation. ^*^P<0.05, ^**^P<0.01 (paired Student's t-test). Table represents sensitizer enhancement ratios for each cell line.

The radiosensitization potential of cisplatin was assessed by clonogenic survival assays (Figure 1 and Supplementary Figure 1).. Three MSC preparations and HS68 fibroblasts were treated with 200 or 1000ng/mL cisplatin for 4 hours, followed by photon irradiation 48 hours later. Cisplatin pre-treatment with 200ng/mL resulted in a small but significant reduction in the clonogenic survival of MSCs (*P<*0.01 for MSC1 and MSC2 and *P<*0.05 for MSC3); sensitizer enhancement ratios (SERs) ranged between 1.07 and 1.10 (Figure 1). Pre-treatment with 1000ng/mL cisplatin led to considerably lower clonogenic survival for all tested MSC samples compared to untreated controls (*P<0*.01 for MSC1, *P<0*.05 for MSC2 and MSC3) and pre-treatment with 200ng/mL (*P<0*.05 in MSC1 and MSC3, *P*=0.09 in MSC2). SER values for the higher cisplatin concentration ranged between 1.24 and 1.30, suggesting a dose-dependent radiosensitization of cisplatin in MSC1 and MSC3. In contrast, HS68 fibroblasts exhibited no dose dependency of cisplatin radiosensitization.

### MSC adhesion is maintained after cisplatin-based chemo-radiation

**Figure 2:**
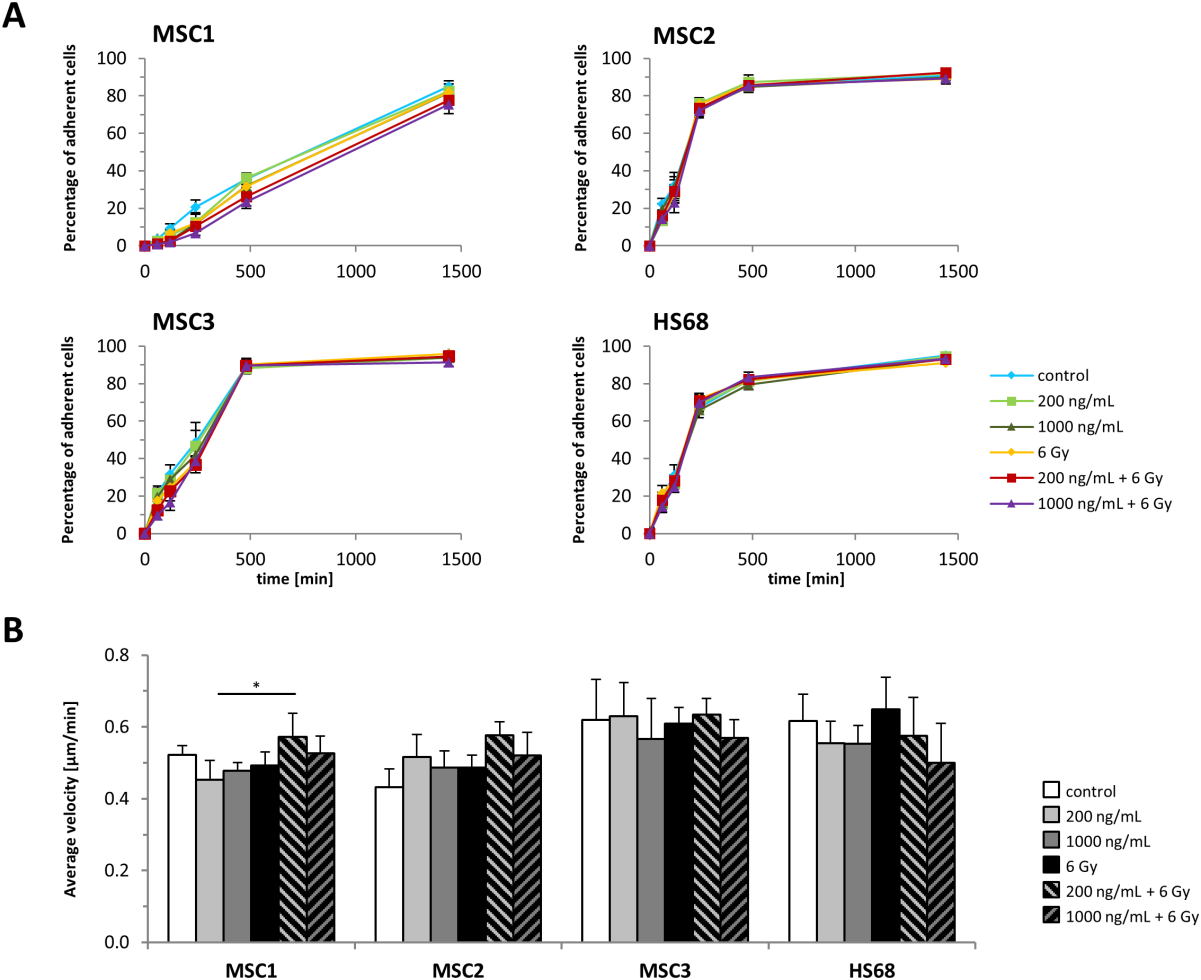
MSC adhesion and motility is unaffected by cisplatin-based chemo-radiation **(A)** Adhesion kinetics of MSCs and HS68 fibroblasts after radiation. **(B)** Average velocity of MSCs and HS68 fibroblasts after treatment with cisplatin, IR or a combined treatment. ^*^P<0.05.

MSC adherence as a defining characteristic of these cells was only insignificantly affected by cisplatin treatment or IR alone (Figure 2A). Similarly, the combination of cisplatin pre-treatment and 6 Gy IR did not affect the adhesion potential of MSC2 and MSC3 preparations compared to untreated controls and only resulted in a small, but significant dose-dependent reduction of MSC1 adherence after irradiation. Differentiated HS68 fibroblasts did not demonstrate reduced adhesion after the combined treatment compared to untreated cells.

**Figure 3:**
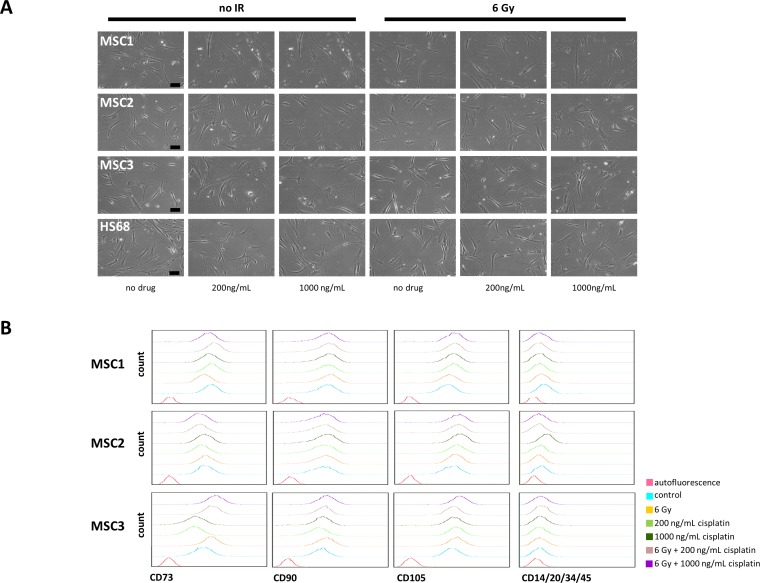
Cisplatin-based chemo-radiation does not alter morphology and surface marker expression of MSCs **(A)** Microscopic images of unstained MSCs and HS68 fibroblasts showing no visible changes in morphology after cisplatin-based chemo-radiation. 10x objective, scale bar 100μm. **(B)** FACS histograms of defining MSC surface markers at 48 hours after cisplatin-based chemo-radiation.

Cellular morphology of MSCs and fibroblasts remained unchanged after combined treatment, and cells revealed no signs of increased apoptosis up to 36 hours by light microscopy (Figure 3A).

### Cisplatin-based chemo-radiation does not impede MSC motility

The average velocity of MSCs as required for cell migration and differentiated fibroblasts was measured by time-lapse microscopy. Neither MSC sample showed a reduction of the average velocity after treatment with cisplatin or IR alone or a combination treatment (Figure 2B). Similarly, the average velocity of HS68 fibroblasts remained unaffected by drug and radiation treatment.

### Cisplatin-based chemo-radiation does not affect MSC surface marker expression

MSC surface markers were examined by flow cytometry at 24 and 48 hours after combined treatment. Protein expression patterns of positive stem cell markers CD73, CD90 and CD105 and negative markers CD14, CD20, CD34 and CD45 remained largely unchanged in all tested samples at 24 (Supplementary Figure 2) and 48 hours after cisplatin-based chemo-radiation (Figure 3B).

### Cisplatin-based chemo-radiation does not abrogate the differentiation potential of MSCs

**Figure 4:**
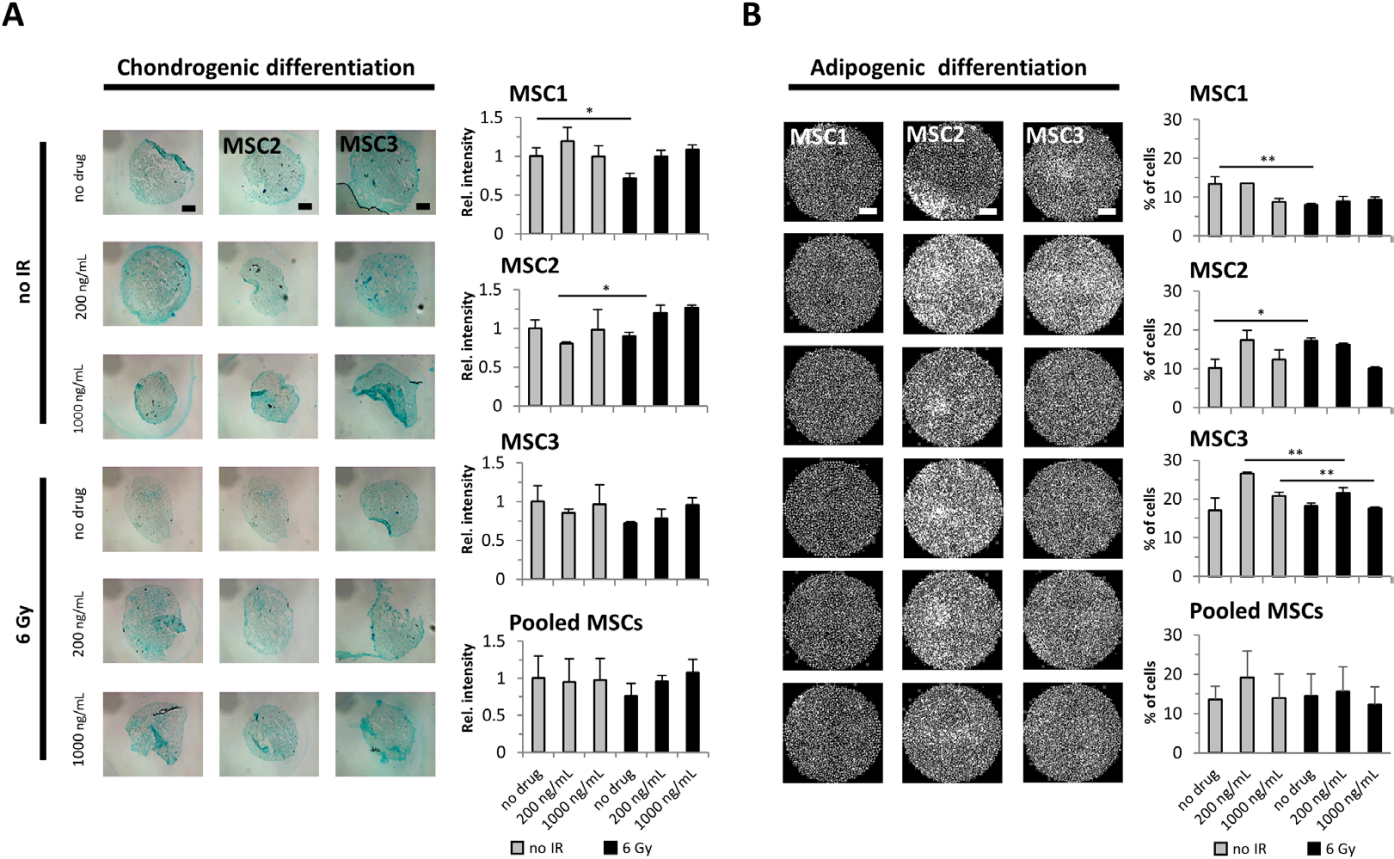
Cisplatin-based chemo-radiation does not affect the differentiation potential of MSCs **(A)** Alcian blue staining for chondrogenic MSC differentiation after treatment with cisplatin and radiation. 2x objective, scale bar 1000μm. **(B)** BODIPY staining for adipogenic differentiation in MSCs. Relative staining intensities were measured to quantify adipogenic and chondrogenic differentiation levels. 2x objective, scale bar 2000μm. ^*^P<0.05, ^**^P<0.01.

The potential for adipogenic and chondrogenic differentiation is a defining hallmark of MSCs. Immunocytochemical analyses were carried out to assess a potential effect of cisplatin-based chemo-radiation on the MSCs’ differentiation ability. Chondrogenic differentiation was not generally altered after treatment with cisplatin or irradiation alone, and only MSC1 samples revealed a small reduction in induced differentiation after 6 Gy radiation (*P<*0.05). Combined treatment with cisplatin and irradiation did not consistently alter the chondrogenic differentiation potential compared to untreated control samples (Figure 4A). Similarly, the ability for adipogenic differentiation was maintained in all MSC preparations after treatment with cisplatin or radiation, and the pooled data revealed no significant reduction after cisplatin-based chemo-radiation compared to untreated cells (Figure 4B).

### Cisplatin pre-treatment shifts MSCs towards the radiosensitive G2/M phase

Cisplatin treatment resulted in a strong accumulation of MSCs in the radiosensitive G2/M phase of the cell cycle with 17 to 58 % in G2/M phase at 48 hours after exposure to 1000ng/mL cisplatin (*P<*0.001 for MSC1 and MSC3, *P<*0.01 for MSC2) (Supplementary Figure 3).

**Figure 5:**
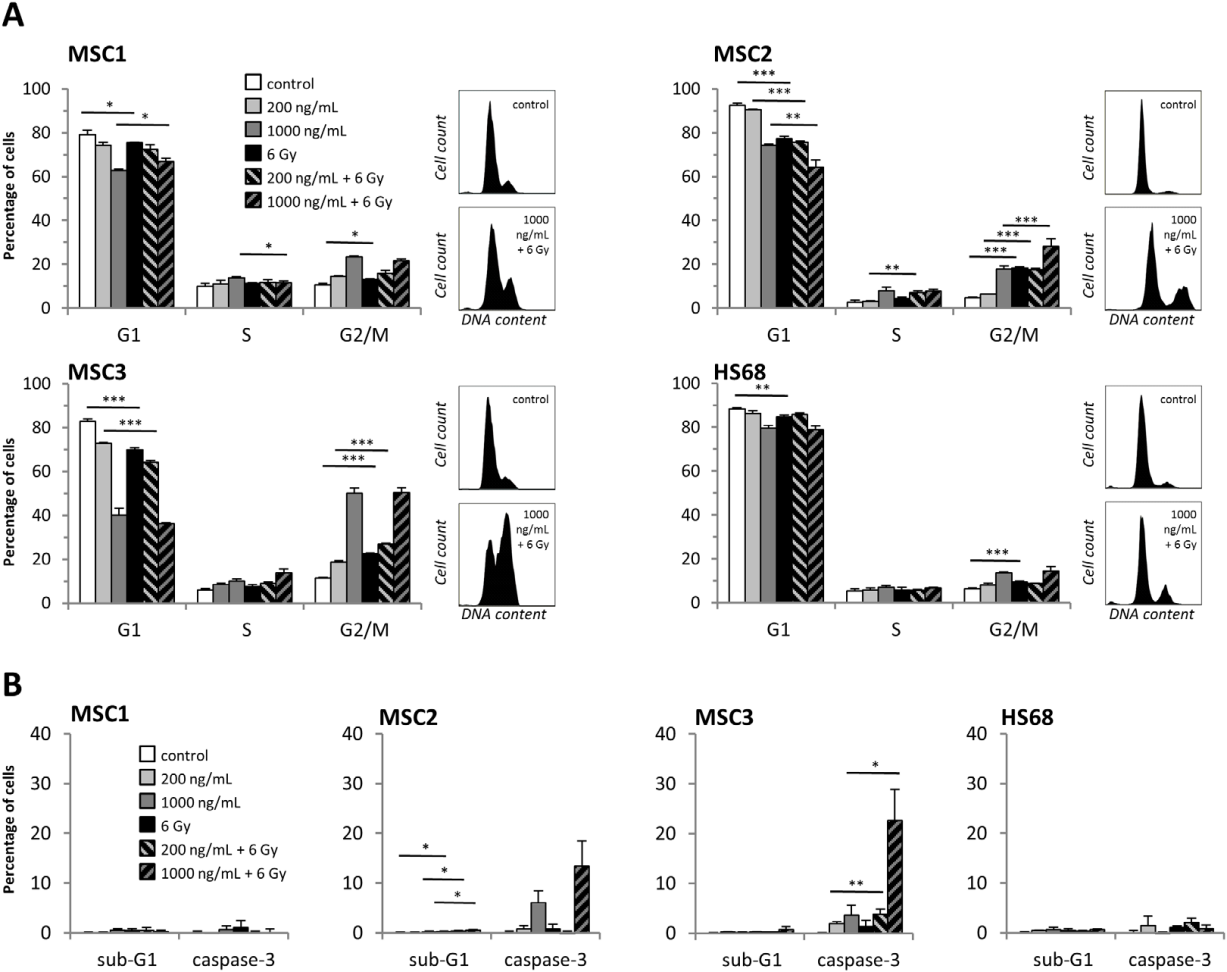
Cisplatin-based chemo-radiation increases apoptosis and G2 phase arrest **(A)** Cell cycle distribution of MSCs and HS68 fibroblasts at 48 hours after cisplatin-based chemo-radiation. **(B)** Apoptosis levels of MSCs and HS68 fibroblasts after cisplatin-based chemo-radiation. ^*^P<0.05, ^**^P<0.01, ^***^P<0.001.

Combining 200ng/mL cisplatin and radiotherapy was found to augment the observed G2 phase block in all three MSC samples compared to treatment with either modality, while pre-treatment with higher doses of 1000ng/mL cisplatin achieved a further increase only in MSC2 (Figure 5A). These findings suggest that cisplatin pre-treatment causes a shift in the cell cycle distribution of MSCs towards the radiosensitive G2/M phase that persists for up to 96 hours after exposure.

HS68 fibroblasts revealed a small increase in their G2 population after exposure to cisplatin or irradiation alone, and a combination of both treatment modalities did not further augment the detected G2 population.

### MSCs show increased apoptosis after cisplatin-based chemo-radiation

Induction of apoptosis after treatment with cisplatin or irradiation was measured by sub-G1, annexin-V staining and caspase-3 activation. While the percentage of sub-G1 cells remained very low independent of cisplatin and radiation treatment, exposure to 1000ng/mL cisplatin resulted in an increase of caspase-3 activation or annexin-V staining in MSC2 and MSC3 samples (Figure 5B, Supplementary Figure 4). Combined treatment with 1000ng/mL cisplatin and 6 Gy radiation led to a further increase in apoptosis in MSC2 and MSC3 cells as detected by caspase-3 activation; in contrast, HS68 fibroblasts did not undergo increased apoptosis following exposure to cisplatin and irradiation.

### Cisplatin pre-treatment increases the number of radiation-induced DNA double-strand breaks in MSCs

**Figure 6:**
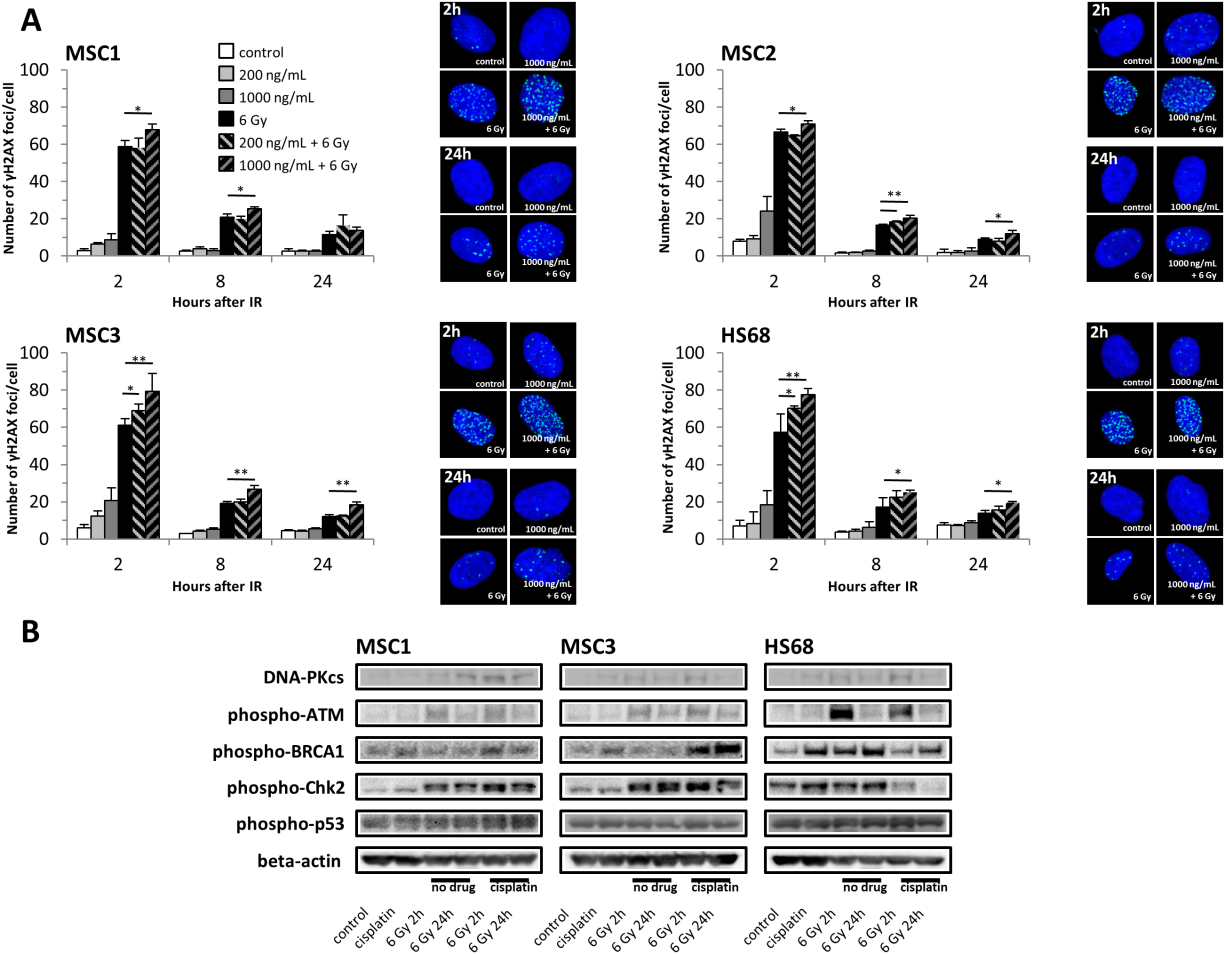
Cisplatin pre-treatment causes prolonged DNA damage signaling and increased radiation-induced DNA double-strand breaks in MSCs **(A)** γH2AX foci numbers in MSCs and HS68 fibroblasts after cisplatin-based chemo-radiation. ^*^P<0.05, ^**^P<0.01. **(B)** Western blot analyses of various proteins regulating cell cycle checkpoints and double-strand DNA break repair at 2 and 24 hours after irradiation with 6 Gy.

Cellular irradiation induces various forms of DNA damage, with DNA double-strand breaks forming the main cytotoxic lesions. To measure the influence of cisplatin pre-treatment on the creation and repair of DNA double-strand breaks, phosphorylated H2AX (γH2AX) foci were quantified as markers for these double-strand breaks. Irradiation resulted in an initial increase in γH2AX foci within 2 hours, but foci levels representing unrepaired strand breaks at 8 and 24 hours after treatment were strongly reduced in all three MSC samples (Figure 6A). While exposure to different concentrations of cisplatin alone did not markedly increase γH2AX foci levels, pre-treatment with 1000ng/mL cisplatin significantly augmented the numbers of initial radiation-induced double-strand breaks in all tested MSC preparations. Lower cisplatin concentrations of 200ng/mL did not increase radiation-induced γH2AX foci levels in MSC1 and MSC2 and were found to increase double-strand breaks only in MSC3 samples, indicating a dose-dependent effect.

Radiation-induced DNA double-strand breaks remained elevated over time in MSCs pre-treated with 1000ng/mL cisplatin: γH2AX foci levels were significantly elevated in all tested MSC specimens after 8 hours and remained elevated up to 24 hours.

Similar results were obtained for differentiated fibroblasts with a significant increase in γH2AX foci in cisplatin-pre-treated cells at all tested time points compared to cells that were only irradiated. These findings suggest that cisplatin pre-treatment increased both initial and residual numbers of DNA double-strand breaks induced by sequential irradiation.

### Cisplatin pre-treatment activates radiation-induced DNA damage recognition pathways in MSCs

DNA damage-signaling pathways were investigated by Western blot analyses. The ataxia-telangiectasia mutated (ATM) kinase and the DNA-dependent protein kinase (DNA-PKcs) as key signaling proteins for homologous recombination repair and non-homologous end-joining repair respectively were found elevated after irradiation irrespective of cisplatin pre-treatment (Figure 6B). In line with the observed persistent γH2AX-labelled DNA double-strand breaks at 24 hours after irradiation, activated phospho-ATM and phospho-BRCA1 involved in the homologous recombination repair of radiation-induced DNA damage remained elevated up to 24 hours, also suggesting residual strand breaks. Consistently, the ATM-dependent checkpoint kinase 2 (Chk2) was found strongly activated after cisplatin-based chemo-radiation, while there was no pronounced effect on phospho-p53, further explaining the observed arrest of treated MSCs in G2, but not G1 phase of the cell cycle.

## DISCUSSION

Cisplatin is widely combined with radiotherapy for cancer treatment. Despite extensive studies, the exact mechanisms by which cisplatin and IR interact remain incompletely understood. Our analyses indicated that cisplatin pre-treatment sensitized radioresistant human mesenchymal stem cells without affecting their defining stem cell characteristics.

Several publications demonstrated a relative resistance of MSCs against IR [19–21] that was linked to an efficient repair of radiation-induced DNA double-strand breaks [22, 23] and the evasion of apoptosis [24, 25]. The defining stem cell characteristics of MSCs were found largely unaffected by irradiation [19, 22, 26]. A similar resistance of MSCs against cisplatin treatment was found associated with high levels of protective heat-shock proteins, cytoskeletal modifications and suppressed apoptosis [27–29].

In our dataset, combining cisplatin and irradiation resulted in a significant dose-dependent radiosensitization of MSCs. A previous study reported a pronounced radiosensitization of mouse fibroblasts by pre-treatment with 1000ng/mL cisplatin, while higher concentrations up to 6000 ng/mL showed reduced SER values compared to the lower dose [30]. We found comparably high SERs in MSCs after pre-treatment with 1000ng/mL, while lowering the cisplatin dose did not cause further increases in SER values.

While the radiosensitizing properties of cisplatin may depend on the treatment scheduling, experimental data investigating the optimal timing of both modalities produced controversial results. In mouse tumors, cisplatin application before irradiation resulted in higher levels of radiation-mediated tumor control than after radiotherapy, while *in vitro*, simultaneous treatment elicited the strongest supra-additive effects [31, 32]. In our dataset, only cisplatin pre-treatment resulted in MSC radiosensitization, while concomitant or post-irradiation treatment with cisplatin did not radiosensitize MSCs. As cisplatin has been shown to accumulate in the body up to several months after exposure, cisplatin pre-treatment may also exert prolonged effects clinically when subsequent radiotherapy is applied [33, 34].

The mechanisms underlying a cisplatin-mediated radiosensitization remain incompletely understood. Cellular radiosensitivities vary according to the cell cycle phase, and G2/M phase cells exhibit the highest sensitivities [35, 36]. Accordingly, we found that cisplatin pre-treatment strongly shifted the MSCs’ cell cycle distribution towards the G2/M phase, resulting in increased radiation-induced apoptosis. Additionally, it has been suggested that cisplatin DNA adducts hamper the repair of radiation-induced DNA lesions, leading to the transformation of sublethal into lethal radiation damage [4]. Previous publications demonstrated efficient repair of DNA damage caused by irradiation or cisplatin alone in MSCs [22, 23, 37]. However, our data revealed that cisplatin pre-treatment increased the number of initial and residual radiation-induced DNA double-strand breaks in MSCs. It has been suggested that γH2AX-labelled double-strand breaks may serve as valuable biomarkers to quantify cellular radiosensitivity, and unrepaired DNA double-strand breaks at 24 hours after irradiation have been linked to an increased cellular radiation sensitivity [38, 39]. In our dataset, cisplatin pre-treatment increased residual double-strand breaks and resulted in prolonged DNA damage signaling up to 24 hours after treatment; hence, the observed increase in unrepaired radiation lesions may help to explain the enhanced MSC radiosensitivity.

Interestingly, despite the observed radiosensitization of MSCs by cisplatin, the surviving stem cells remained functionally unaffected. Of note, the differentiation potential as the basis for the reported regenerative effects of MSCs remained largely intact after cisplatin-based chemo-radiotherapy. This finding may have special clinical relevance, as several publications provided evidence that endogenous MSCs could invade into tissue lesions caused by cisplatin or IR and could differentiate into functional cell types, thereby aiding tissue regeneration [24, 40–44].

While the observed cisplatin-based radiosensitization may not directly affect the treatment of chemo-radiation-induced side effects with exogenous MSCs, such an application would require a concomitant immunosuppression to avoid graft rejection. It is therefore conceivable that endogenous MSCs may be utilized in the future to treat tissue damage caused by cisplatin-based chemo-radiation: Stimulation or harvesting and *ex-vivo* proliferation of endogenous MSCs after the occurrence of chemo-radiation-induced tissue lesions may provide an on-demand cell-based therapeutic approach to treat those side effects. However, as the use of these endogenous MSCs is directly dependent on their ability to survive after cisplatin-based chemo-radiation, the observed increase in cisplatin-mediated MSC radiosensitization needs to be taken into consideration when planning treatment protocols utilizing endogenous MSCs for chemo-radiotherapy-associated side effects (18,43). On the other hand, the data shown here may help to explain the augmented severe late toxicities often seen after combining cisplatin and radiotherapy for routine treatments, e.g. in head and neck or cervical cancers: Since the endogenous regeneration of tissue damage induced by cisplatin-based chemo-radiation in part depends on the activation of organ-resident MSCs, the reported increase in MSC radiosensitization by cisplatin may result in reduced numbers of regenerative tissue-resident stem cells by the combined treatment and hence in impaired tissue regeneration [45, 46].

Taken together, our data demonstrated an increased radiation sensitivity of MSCs after pre-treatment with cisplatin, whereas their stem cell properties were largely maintained. A cisplatin-mediated shift in the cell cycle distribution and an increase in residual DNA double-strand breaks may contribute to the radiosensitizing potential of cisplatin in MSCs.

## MATERIALS AND METHODS

### Cell culture

MSCs were isolated from bone marrow of healthy volunteers and characterized as published previously [47, 48]. MSCs were cultured in Mesenchymal Stem Cell Growth Medium containing *MSCGM™* SingleQuots (*MSCGM™*, Lonza, Basel, Switzerland). Human HS68 dermal fibroblasts were purchased from the ATCC and grown in Dulbecco's Modified Eagle Medium (Biochrom, Berlin, Germany) with 10% fetal bovine serum and 3.5g/L glucose. Donors provided written informed consent prior to the harvesting of MSCs, and this work was approved by the Heidelberg University ethics board (#S-384/2004).

### Drug preparation

Cisplatin solution was received from the Heidelberg University Hospital central pharmacy and was kept refrigerated for a maximum of 7 days for the use in this study. The drug was diluted to either 200 or 1000ng/mL immediately prior to its use. All experimental setups containing cisplatin were protected from light.

### Clonogenic survival assays

Between 1000 (0 Gy) and 8000 (6 Gy) cells were plated and allowed to attach before treatment. Cisplatin treatment was carried out at concentrations of 200 or 1000ng/mL for 4 hours. Irradiation was performed using a 6 MeV linear accelerator. Timing of drug treatment and irradiation for each experiment is indicated in the respective figure legends. After 14 days, colonies were fixed with 25% acetic acid (v/v) in methanol and stained using crystal violet solution. Colonies exceeding 50 cells were counted, and the cellular surviving fraction was calculated according to the following formula: (#colonies/#plated cells)_treated_/(#colonies/#plated cells)_untreated_. Plating efficiencies were 6.53±2.65% for MSC1, 6.79±1.41% for MSC2, 3.58±1.98% for MSC3 and 6.11±0.33% for HS68 cells. The sensitizer enhancement ratio (SER) for cisplatin was calculated at 10% survival; radiosensitization was assumed for SER values >1.1. Survival curves were modeled according to the linear-quadratic model using Sigma Plot version 13 (SyStat Software, San Jose, USA). All experiments were carried out in triplicate, and statistical comparison of survival curves was performed using paired Student's t-tests.

### Cell adhesion measurements

Cells were exposed to 200 or 1000ng/mL cisplatin for 4 hours and incubated for 48 hours before irradiation. 100 cells were transferred to each well of 96-well plates, and attached cells over time were evaluated by light microscopy. The attachment efficiency was calculated as the ratio between attached and plated cells.

### Cell motility measurements

10 000 cells were plated in each well of a 24-well plate and treated with 200 or 1000ng/mL cisplatin for 4 hours, followed by irradiation. Movement of MSCs and fibroblasts was investigated at 7-minute intervals over 35 hours by time-lapse microscopy. Images were acquired on an IX70 microscope (Olympus, Hamburg, Germany). Migratory tracks of treated cells were quantified using ImageJ software (National Institutes of Health, Bethesda, USA).

### Surface marker expression

MSCs were pre-treated with 200 or 1000ng/mL cisplatin and irradiated 48 hours later. At 24 and 48 hours after radiotherapy, surface marker expression was analyzed on a FACSCanto flow cytometer (Becton-Dickinson, Heidelberg, Germany)[17]. Surface marker stainings were performed using a MSC phenotyping kit according to the manufacturer's instructions (Miltenyi Biotec, Bergisch-Gladbach, Germany), and 10,000 events were recorded for each treatment condition before data analysis with FlowJo 7.6.5 software (FlowJo LLC, Ashland, USA).

### MSC differentiation

MSCs were treated with cisplatin for 4 hours and exposed to 6 Gy IR 48 hours later. Culturing medium was then replaced with differentiation media, and cells were proliferated for 21 days.

Chondrogenic differentiation was induced using the STEMPRO® Chondrogenesis Differentiation Kit (Gibco Life Technologies, Frankfurt, Germany). MSC spheroids were fixed with 4% paraformaldehyde solution before sectioning on a cryomicrotome. Staining was carried out with 1% Alcian Blue solution for 30 minutes at room temperature.

Adipogenic differentiation was induced using DMEM containing 10% FCS, 2mM L-glutamine, 1μM dexamethasone, 500μM 1-methyl-3-isobutylxanthine, 1μg/mL insulin and 100U/mL penicillin/streptomycin. Differentiated cells were stained in 1μM/mL BODIPY (493/503) solution for 20 minutes (Life Technologies, Darmstadt, Germany) and nuclei were counterstained with 1μM 4′,6-diamidin-2-phenylindol (DAPI). Fluorescence images were taken on a Keyence BioRevo9000 microscope, and mean fluorescence intensities were quantified using ImageJ software.

### Cell cycle analysis and apoptosis measurements

Cells were treated with cisplatin for 4 hours followed by irradiation 48 hours later. At 24 and 48 hours after treatment, cells were fixed in 3% paraformaldehyde solution, permeabilized in ice-cold 70% ethanol and incubated with a fluorescence-coupled antibody against activated caspase-3 (1:20, BD Pharmingen, Heidelberg, Germany) for 1 hour at room temperature. Nuclei were counterstained with 1μg/mL DAPI solution. Flow cytometry measurements were carried out on a LSR-II system, recording 10,000 events for each experimental condition. Early apoptosis was measured by annexin-V staining with the FITC Annexin-V Apoptosis Detection Kit (BioLegend, San Diego, USA) according to the manufacturer's instructions. Data analysis was performed using FlowJo 7.6.5 software.

### Analysis of DNA double-strand break foci

10 000 cells were plated in 24-well plates before a 4-hour pre-treatment with cisplatin and irradiation. Cells were fixed with 4% paraformaldehyde solution and permeabilized with ice-cold 70% ethanol before incubation with a mouse monoclonal antibody against γH2AX (1:100, Biolegend, London, UK) and an Alexa Fluor-488-coupled goat anti-mouse antibody (1:250, Invitrogen, Darmstadt, Germany) at 4°C. Nuclei were stained with DAPI for 5 min, and images were taken at 40x magnification on an Axioplan2 microscope (Zeiss, Jena, Germany). For each treatment condition, 300 cells were automatically detected, and foci were counted using Metafer software (Metasystems, Altlussheim, Germany).

### Western blot analyses

Western blots were performed as reported previously [49]. In short, cells were pre-treated with 1000 ng/mL cisplatin for 4 hours and irradiated 48 hours later before harvesting at 2 and 24 hours after irradiation. Protein samples were run on polyacrylamide gels and transferred to polyvinylidene difluoride membranes (Millipore, Darmstadt, Germany). Membranes were probed with antibodies against phospho-Chk2 (1:1000, Cell Signaling Technology, Leiden, Netherlands), phospho-p53 (1:1000, Cell Signaling Technology), phospho-ATM (1:1000, R&D, Wiesbaden, Germany), phospho-BRCA1 (1:1000, Cell Signaling Technology) and DNA-PKcs (1:1000, Cell Signaling Technology). β-actin was used as a loading control (1:2000, Cell Signaling Technology). Blots were visualized on X-ray film using a horseradish-peroxidase kit (Cell Signaling Technology).

## SUPPLEMENTARY FIGURES


