# Supplementary material for: Cisplatin radiosensitizes radioresistant human mesenchymal stem cells
Source: Oncotarget. 2017 Sep 23;8(50):87809–20. doi: 10.18632/oncotarget.21214 (PMC5675674; doi:10.18632/oncotarget.21214)
Supplement: Supplementary file 1 [file oncotarget-08-87809-s001.pdf]

Cisplatin radiosensitizes radioresistant human mesenchymal stem cells

SUPPLEMENTARY MATERIALS

A

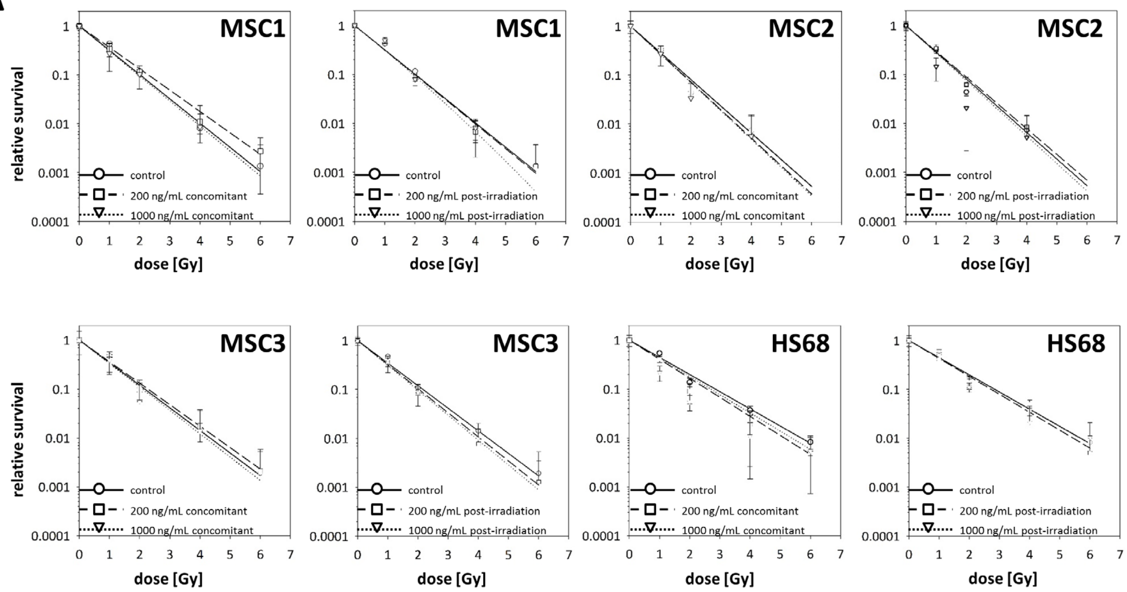

B

Cisplatin dose	MSC1	MSC2	MSC3	HS68
**200 ng/mL concomitant**	0.88	1.05	0.95	1.11
**1000 ng/mL concomitant**	1.02	1.06	1.04	1.06
**200 ng/mL post-irradiation**	1.01	0.97	1.07	1.05
**1000 ng/mL post-irradiation**	1.04	1.03	1.10	1.00

Supplementary Figure 1: Post-irradiation or concomitant cisplatin treatment does not lead to radiosensitization. (A) Clonogenic survival assays of MSCs and HS68 fibroblasts after concomitant cisplatin and radiation treatment (“concomitant”) or treatment with cisplatin 24 hours after irradiation (“post-irradiation”). **(B)** SER values for each cell line and treatment scheduling.

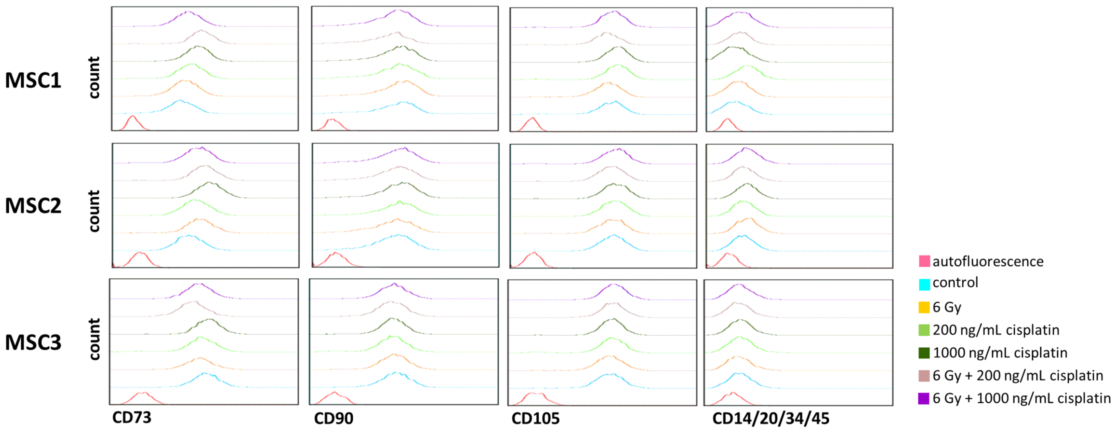

Supplementary Figure 2: MSC surface marker expression is unaffected by cisplatin-based chemo-radiation. Flow cytometry analyses of positive and negative MSC surface markers at 24 hours after cisplatin-based chemo-radiation.

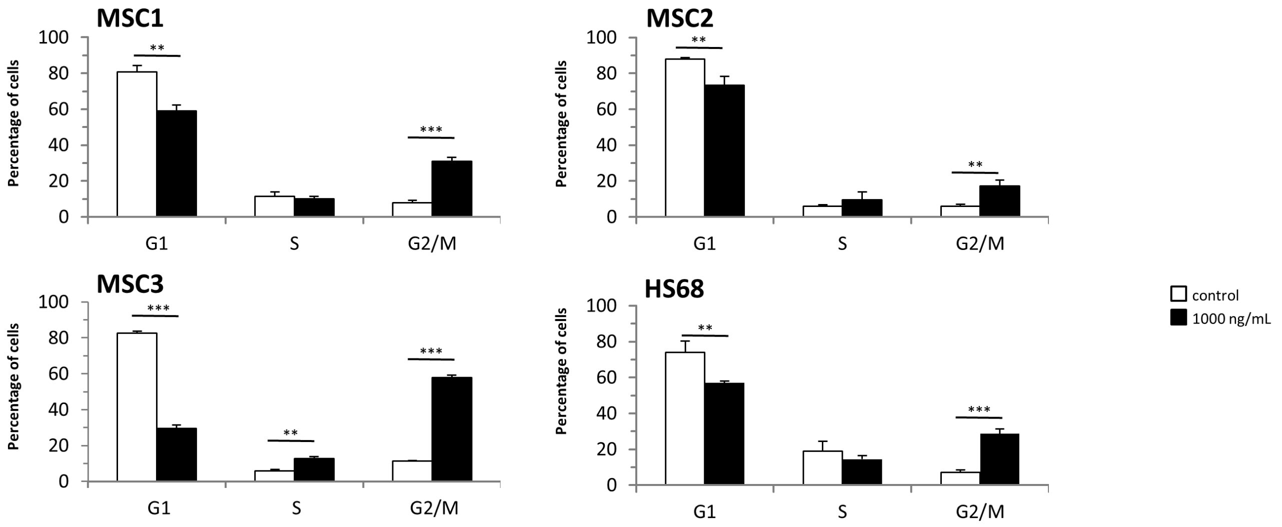

Supplementary Figure 3: Cisplatin pre-treatment results in a G2 phase accumulation. Cell cycle distribution of MSCs and HS68 fibroblasts at 48 hours after exposure to 1000ng/mL cisplatin. $^{**}P<0.01$, $^{***}P<0.001$.

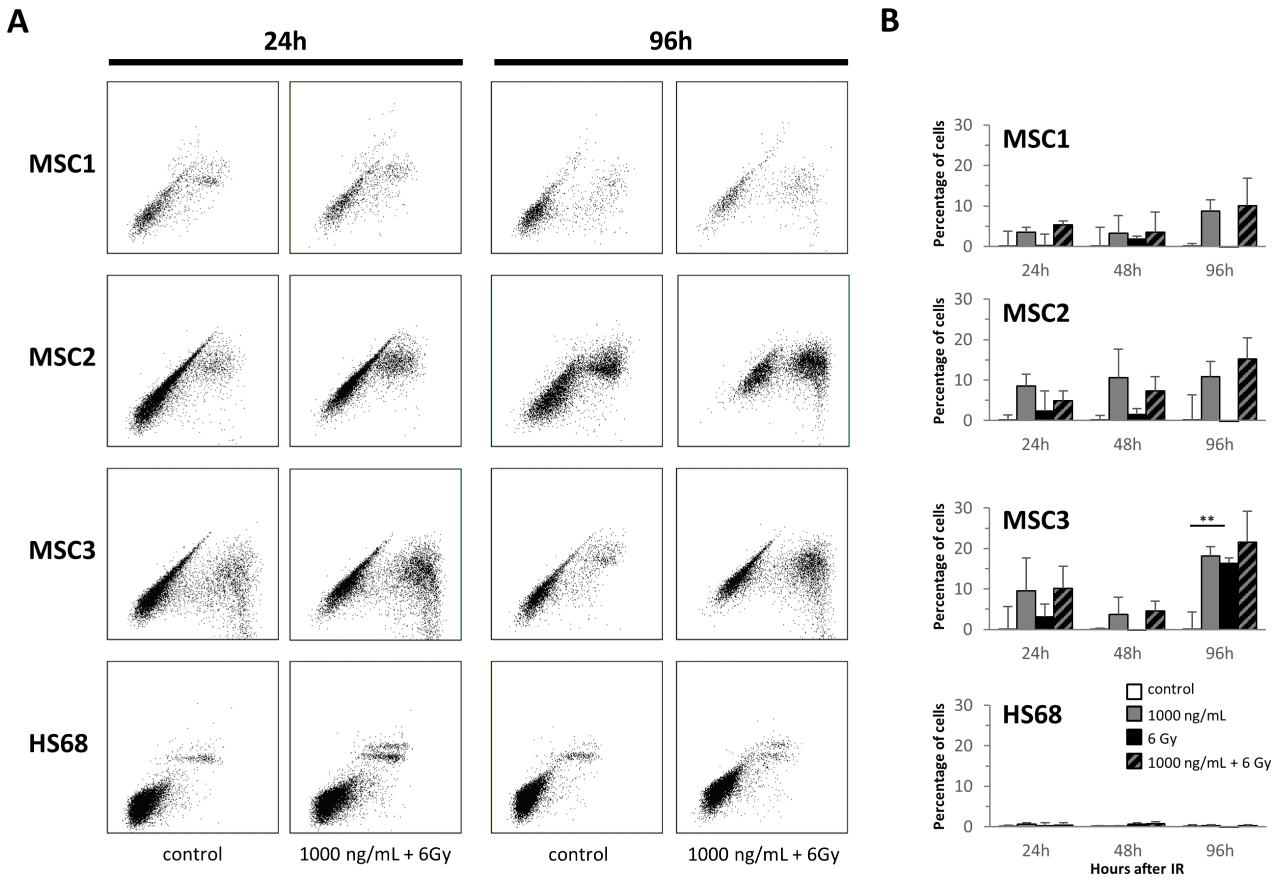

Supplementary Figure 4: Cisplatin-based chemo-radiation increases early apoptosis in MSCs. **(A)** Dot plots of simultaneous annexin-V and 7-AAD staining at 24 and 96 hours after cisplatin-based chemo-radiation. **(B)** Early apoptosis levels of MSCs and HS68 fibroblasts at various time points after cisplatin-based chemo-radiation. ***P<0*.01.
